# Case Report: Treatment of Severe Subcutaneous Emphysema With a Negative Pressure Wound Therapy Dressing

**Published:** 2009-01-07

**Authors:** Christopher M. Sciortino, Gerhard S. Mundinger, David P. Kuwayama, Stephen C. Yang, Marc S. Sussman

**Affiliations:** ^a^ Department of Surgery, Johns Hopkins Bayview Medical Center, Baltimore, Md.; ^b^ Division of Plastic, Reconstructive, and Maxillofacial Surgery, Johns Hopkins Bayview Medical Center, Baltimore, Md.; ^c^ Division of Thoracic Surgery, Johns Hopkins Bayview Medical Center, Baltimore, Md.

## Abstract

**Objective:** This article describes a patient who developed severe subcutaneous emphysema and a persistent air leak after several attempts at needle thoracostomy for what was thought to be a tension pneumothorax. Subcutaneous emphysema was effectively treated with a topical negative pressure wound therapy dressing applied to a typical subfacial “blowhole” incision. This article aims to describe and contextualize the use of negative pressure wound therapy within the existing treatment options for subcutaneous emphysema. **Methods:** A case report of the clinical course and technique was drafted, and the relevant literature in PubMed was reviewed. **Results:** The level of subcutaneous emphysema decreased significantly within 48 hours of negative pressure wound therapy as confirmed with physical examination and computed tomography scans. Negative pressure wound therapy for subcutaneous emphysema has not been previously described in the literature. **Conclusions:** Negative pressure wound therapy applied over subfascial incisions is a novel technique that effectively and rapidly controlled massive subcutaneous emphysema and persistent air leak. This technique may be efficacious in other cases of subcutaneous emphysema.

## CASE REPORT

The patient is a 70-year-old African American man with a history of bullous emphysema who presented with a 3-week history of severe left-sided chest pain to the emergency department. He had undergone a left upper lobe bullectomy at an outside associated institution 1 month prior to presentation for macrobullous emphysema with 4 prior spontaneous pneumothoraces. Chest computed tomography scan in the emergency department was unremarkable with no signs of pneumothorax, pulmonary embolism (PE), or pneumonia. Results from electrocardiographic and routine laboratory studies were within normal limits. The patient was admitted to the medical service for pain control and started on a patient controlled analgesia pump.

**Figure 1:**
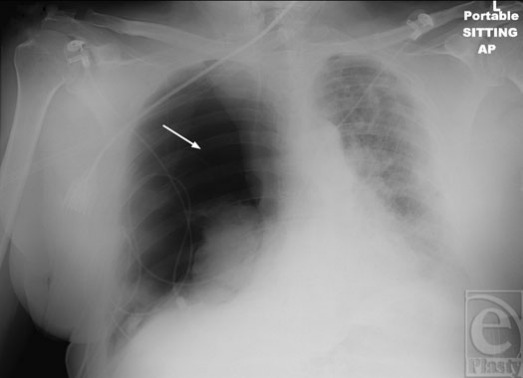
Portable roentogram obtained shortly after the acute onset of chest pain, tachypnea, and desaturation. Significant pneumothorax is evident (white arrow). Multiple attempts at needle decompression were performed without success prior to chest tube insertion.

Approximately 48 hours after admission, the patient experienced an acute increase in chest pain and became tachycardic, tachypneic, and desaturated to 60% by pulse oximeter on nonrebreather oxygen supplementation. He was emergently intubated and transferred to the medical intensive care unit. Portable roentogram revealed a large right-sided pneumothorax (Fig 1). Three attempts at right needle thoracostomy were performed without a notable rush of air or change in hemodynamics.

**Figure 2:**
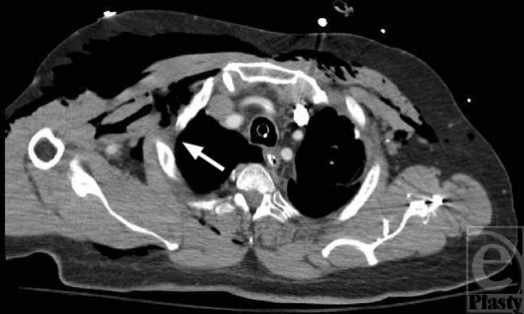
Computed tomography imaging at the second intercostal space shortly after chest tube insertion demonstrating subcutaneous emphysema due to persistent air leak originating from sites of repeated needle thoracostomy attempts (white arrow). The air leak was exacerbated by high peak airway pressures required to maintain oxygenation and ventilation.

**Figure 3:**
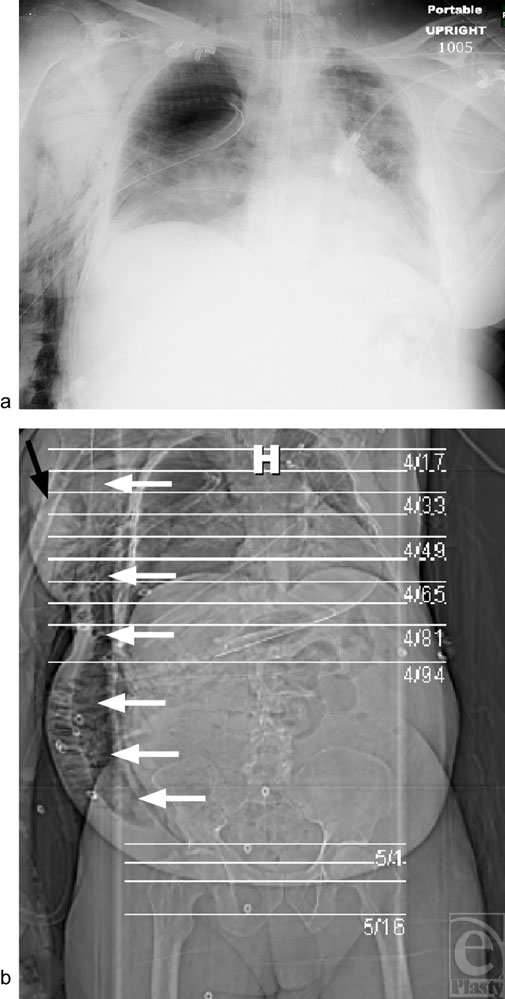
(*a*) Portable roentogram shortly after right tube thoracostomy following repeated unsuccessful attempts at needle thoracostomy demonstrates rapid development of extensive subcutaneous emphysema in conjunction with positive pressure ventilation and persistent chest tube air leak. (*b*) Computed tomography scout film approximately 12 hours after tube thoracostomy demonstrates wide extension and severe degree of subcutaneous emphysema (white arrows). Prepectoral “blowhole” incision (black arrow).

The thoracic surgery team was emergently consulted for chest tube placement. An air rush was noted upon entry in to the right hemithorax and the patient stabilized after chest tube placement. A sizable continuous air leak was noted upon chest tube insertion, as was a considerable amount of right anterior chest subcutaneous emphysema (SE) that tracked inferiorly from the infraclavicular sites of repeated attempts at needle thoracostomy (Figs 2 and 3*a*). Of note, the patient required high amounts of ventilator positive end-expiratory pressure to maintain adequate oxygenation. A 2-cm “blowhole” incision was made below the right clavicle through the skin and prepectoral fascia in an attempt to allow subcutaneous plethora decompression.^1^

**Figure 4:**
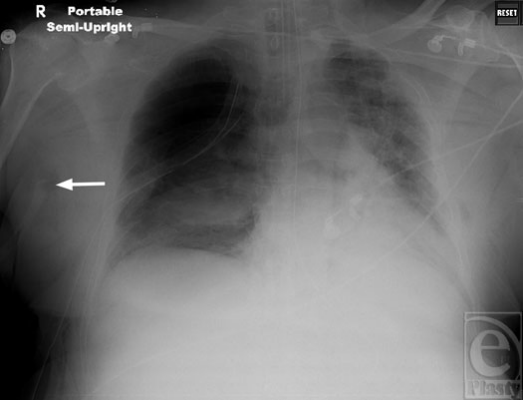
Portable roentogram demonstrates resolution of subcutaneous emphysema approximately 48 hours following application of topical negative pressure wound therapy dressing (white arrow).

**Figure 5:**
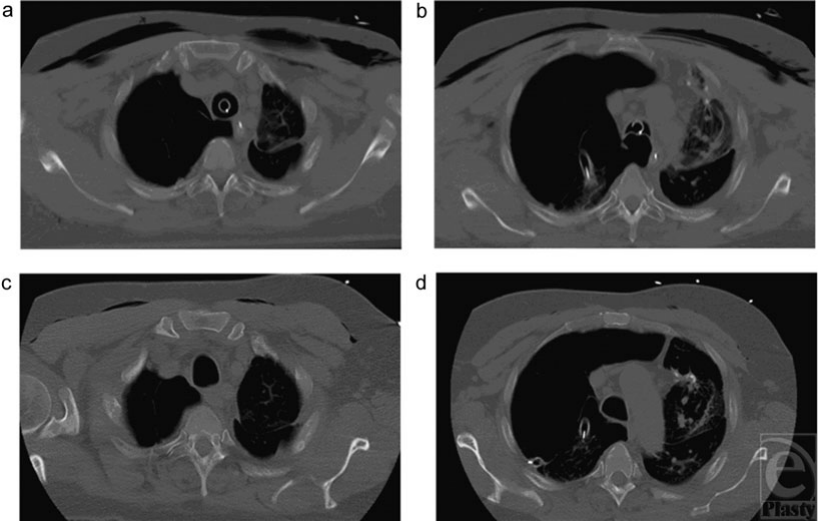
Representative saggital computed tomography scan images of the chest at the level of the sternoclavicular joint (*a*) and the top of the aortic arch (*b*)taken shortly after tube thoracostomy. Note the extensive bilateral subcutaneous emphysema. Corresponding sections after 48 hours of NPWT (*c*, *d*, respectively) demonstrate near-complete resolution of SE.

No appreciable change in body contour or crepitance was noted over the next 48 hours; however, the patient's severe SE, which had tracked inferiorly into the abdominal subcutaneous tissues, had stabilized (Fig 3*b*). A topical negative pressure wound therapy (NPWT) dressing (VAC dressing, KCI International, San Antonio, Tex) was inserted into the blowhole incision and set at a continuous suction of 100 mm Hg (Fig 4). Over the subsequent 48 hours. there was near-complete resolution of SE as supported by physical examination and repeat computed tomography scan (Fig 5). Over the ensuing 4 days, the patient stabilized and was ultimately extubated. An air leak persisted but was adequately controlled by the chest tube. The patient underwent definitive right upper lobe bullectomy approximately 3 weeks later to remove bullae ruptured during attempted needle decompression and chest tube placement. He was discharged in good condition on postoperative day number 6 after a right thoracotomy and right upper lobe bullectomy.

## DISCUSSION

Subcutaneous emphysema is a well-recognized and infrequent occurrence in critically ill patients from various causes and an uncommon complication following pulmonary resection or airway procedures either via thoracotomy or video-assisted thoracoscopic surgery (VATS).^2^ It may also develop after chest tube placement, or as in this case, with needle decompression of the thorax for tension pneoumothorax, especially if the lung parenchyma is violated. The mechanism of SE development is air escaping from the thorax into the subcutaneous and subfacial spaces as a result of pulmonary parenchymal disruption.^2^

Clinical manifestations of SE vary widely depending on its severity and extent. Patients may notice mild (and temporary) disfigurement of their body contour or experience mild to moderate pain. SE can track to the neck and result in stridor or life-threatening compression of the trachea.^3^ SE can additionally track to the orbits and compress the globe, threatening vision.^4^,^5^

The main objective of SE treatment is controlling the source of air escaping into the subcutaneous space, which allows the proper inflation of the lung, apposition of the pleura surfaces, and subsequently injured parenchyma to reapproximate and heal. Standard treatment involves chest tube placement in an optimal position adequate to evacuate the majority of escaping air. Management options for SE can then focus on evacuation of subcutaneous air. Treatment modalities include observation, tissue massage, incising the skin and subcutaneous fascia to create a “blowhole” to allow air to escape, fenestrated angiocatheter insertion in to the subcutaneous space, and VATS or open thoracotomy with repair of parenchymal injury.^1^–^3^,^6^–^11^

The time to SE resolution varies greatly depending on the amount of SE, adequacy of control of escaping air, and method of treatment. There is a paucity of scientific studies comparing different SE treatment modalities. With observation alone, in which air is gradually reabsorbed by soft tissues, it can take several weeks for significant SE to resolve. A “blowhole” incision is widely utilized to allow the release of trapped subcutaneous air and to minimize further progression of air dissection into the face and neck. In this method, a small (approximately 2 cm) incision in the infraclavicluar region through the skin and prepectoral fascia is made at the bedside under local anesthesia.^1^ This method is frequently used at our institution and relies on the passive diffusion of trapped air out of the incision. The wound is typically packed with gauze and the dressing is changed twice daily. Blowhole placement can be effective in decreasing tense SE over the course of several days; however, complete resolution in cases of significant SE can take a week or more. A variety of microcatheter suction techniques have been described to evacuate SE.^7^–^11^ These methods utilize fenestrated angiocatheters or drains placed subcutaneously and set to continuous suction and can significantly reduce SE in several days. A more invasive method of SE control requires operative management and control of the source of escaping air. A prospective trial by Cerfolio et al^6^ has shown VATS to be an excellent means of controlling persistent air leak.

In this case, the extensive and rapidly expanding SE was due to a large and persistent communication between the pleural and subcutaneous space following parenchymal tears resulting from repeated needle decompression. This communication was exacerbated by the need for positive pressure ventilation with high positive end expiratory pressure (PEEP). We felt that, given the massive extent of SE and persistence of air leak (Figs 2 and 3), previously described means of microcatheter evacuation would likely fail. Definitive surgical treatment of the underlying parenchymal injury was felt to be a poor immediate option, as the patient, now stabilized, was clearly a high-risk surgical candidate. While tracheal compression was not imminent, there was concern that it could develop given the size of the air leak and rapid SE expansion. We therefore considered novel treatment means that would provide SE evacuation adequate to control the persistent air leak.

NPWT dressings have been utilized in a wide variety of clinical settings beyond simple wound care and clinical indications for their use continue to broaden.^12^–^14^ These devices have demonstrated great utility in decreasing edema via a negative pressure gradient that pulls fluid out of wounds.^12^,^15^–^18^ We postulated that since trapped air in the subcutaneous and subfascial space was in continuity with the source of air escaping from sites of attempted needle decompression, the placement of an occlusive suction dressing would allow for aspiration of the trapped air. Clinically, NPWT potentiated rapid resolution of both SE and air leak.

Several important parameters need to be addressed prior to NPWT placement for SE. Adequate control of the underlying air leak, if present, is key to resolution of lung parenchyma injury and cessation of air escape from the thorax. NPWT does not replace tube thoracostomy in these cases; it is an adjunct to therapy for SE once a chest tube has been placed. An open chest wound should be considered a contraindication to the NPWT technique. Other considerations such as increased wound care needs, potential for increased wound site pain, concerns about development of infection at the wound site, equipment costs, and dressing availability must be carefully evaluated prior to initiation of NPWT for SE. Although not observed in this case, increasing NPWT suction required to maintain an adequate seal may indicate a disruption in the chest tube circuit or a larger air leak from the lung, and the treating physician should be mindful of this potentiality throughout the duration of treatment. With cognizance of these parameters, a vacuum dressing can likely be applied in most cases where a “blowhole” incision is deemed necessary to potentiate rapid evacuation of SE. SE is another clinical scenario in which NPWT may be an effective management option.

## SUMMARY

Topical NPWT for evacuation of SE has not been previously described in the literature. We report a case in which NPWT was rapidly efficacious in evacuating massive SE and sealing a persistent air leak following repeated attempts at needle decompression for tension pneumothorax. NPWT may be applicable to most cases in which evacuation of SE is required and is yet another clinical scenario where this treatment modality is effective.
